# Long-term outcomes of coils embolization for superior hypophyseal artery aneurysms

**DOI:** 10.3389/fneur.2023.1096970

**Published:** 2023-06-29

**Authors:** Yan-Po Kang, Cheng-Yu Li, Chun-Ting Chen, Mun-Chun Yeap, Ho-Fai Wong, Yi-Ming Wu, Po-Chuan Hsieh, Zhuo-Hao Liu, Chi-Cheng Chuang, Ching-Chang Chen

**Affiliations:** ^1^Department of Neurosurgery, Chang Gung Memorial Hospital at Linkou, Chang Gung University College of Medicine, Taoyuan, Taiwan; ^2^Department of Neurosurgery, New Taipei Municipal Tucheng Hospital (Built and Operated by Chang Gung Medical Foundation), New Taipei, Taiwan; ^3^Department of Radiology, Chang Gung Memorial Hospital at Linkou, Chang Gung University College of Medicine, Taoyuan, Taiwan

**Keywords:** stent-assisted coiling, coils embolization, aneurysm recurrence, superior hypophyseal artery, intracerebral aneurysm

## Abstract

**Objective:**

Superior hypophyseal artery (SHA) aneurysms are intradural, and their rupture can result in subarachnoid hemorrhage. Considering the related surgical difficulty and anatomical restrictions, endovascular treatment (EVT) is considered the most favorable modality for SHA aneurysms; however, the long-term outcomes of EVT have rarely been reported. The study assessed the incidence of and risk factors for recurrence of SHA aneurysms after EVT as well as the correlation factors for SHA aneurysm rupture.

**Methods:**

We included 112 patients with SHA aneurysms treated with EVT at our facility between 2009 and 2020. Here, EVT included non–stent-assisted (simple or balloon-assisted) or stent-assisted coiling. Flow diverter was not included because it was barely used due to its high cost under our national insurance’s limitation, and a high proportion of ruptured aneurysms in our series. Univariate and multivariate logistic regression was performed to evaluate the correlation factors for SHA aneurysm rupture, along with the incidence of and risk factors for post-EVT SHA aneurysm recurrence and re-treatment.

**Results:**

In our patients, the mean angiographic follow-up period was 3.12 years. The presence of type IA or IB cavernous internal carotid artery (cICA) was strongly correlated with SHA aneurysm rupture. Recurrence occurred in 17 (13.4%) patients, of which only 1 (1.4%) patient had received stent-assisted coiling. All cases of recurrence were observed within 2 years after EVT. The multivariate logistic regression results showed that ruptured aneurysm and non–stent-assisted coiling were independent risk factors for aneurysm recurrence. Of the 17 cases of aneurysm recurrence, 9 (52.9%) received re-treatment. Moreover, aneurysm rupture was the only factor significantly correlated with re-treatment in multivariate logistic regression. No re-recurrence was observed when a recurrent aneurysm was treated with stent-assisted coiling.

**Conclusion:**

Type I cICA was common factor for aneurysm rupture. Although flow-diverter treatment serves as another suitable technique that was not compared with, coils embolization was effective treatment modality for SHA aneurysms, leading to low recurrence and complication rates, especially with stent use. All cases of recurrence occurred within 2 years after EVT; they were strongly associated with prior aneurysm rupture. Further stent-assisted coiling was noticed to prevent re-recurrence.

## Introduction

The superior hypophyseal artery (SHA) originates from the internal carotid artery (ICA) between the ophthalmic and posterior communicating (P-com) artery (1). The origin of SHA aneurysms is typically distal to the distal dural ring; therefore, SHA aneurysms are intradural, and their rupture may result in a subarachnoid hemorrhage (SAH) (2). However, most studies have included SHA aneurysms – with clinoidal, carotid cave, and ophthalmic aneurysms – among paraclinoid aneurysms (1, 3, 4), which are considered to have a low rupture rate (5, 6).

Although research on the characteristics and treatment outcomes of endovascular treatment (EVT) for SHA aneurysms has been scant, the rupture risk is higher for SHA aneurysms than for paraclinoid aneurysms (5, 7). Moreover, SHA aneurysm rupture can lead to SAH, which has detrimental clinical outcomes (8). Timely SHA aneurysm treatment is therefore necessary, and so the research article that focuses on SHA aneurysms is worth studying.

In paraclinoid aneurysm treatment, surgical clipping is considered challenging due to the related anatomical restrictions along with the adjacent delicate bony and neurovascular structures (1), which has been found to lead to high complication and mortality rates (9). EVT has consequently become the mainstream treatment modality for paraclinoid aneurysms because of its treatment safety and efficacy (1, 10). In addition, patient preference, short operative time and reducing length of stay have shifted the trend of paraclinoid aneurysm treatment toward this less-invasive technique (11).

Research on SHA aneurysms with respect to aneurysm characteristics, treatment options, and long-term follow-up has been scant. Nevertheless, Chalouhi, Tjoumakaris (7) assessed the safety and efficacy of coils embolization for SHA aneurysms, but its average follow-up period was <1 year (i.e., 10.4 months). Furthermore, the authors’ study was observational and lacked any statistical analysis due to a low event number.

Here, we analyzed the features of SHA aneurysms and identified the predictors for their recurrence and re-treatment after EVT. To the best of our knowledge, this is the largest retrospective case–control study with the longest follow-up duration on EVT for SHA aneurysms. The current results may facilitate the design of personalized EVT.

## Materials and methods

### Patient population

We reviewed our aneurysm database for all patients with SHA saccular aneurysms who underwent EVT from January 2009 to December 2020. Aneurysms associated with fusiform dilatation or dolichoectatic change of the ICA were excluded from this study. In this study, we only focus on endovascular treatment (EVT) because surgical clipping is rarely applied in recent years at our facility since extending the exposure of skull base may damage adjacent neurovascular structures (1), and based on previous literature (7) we believe EVT nowadays has become the mainstream treatment modality that offers safe and satisfactory results. Patients who underwent flow-diverter treatment were also excluded because flow diverter is costly and our national insurance only covers multiple and giant aneurysms regardless of aneurysm location. Also, the nature of numerous ruptured cases in the study made flow-diverter unsuitable for these patients. Finally, 112 patients (23 males and 89 females) who underwent 128 sessions of treatment for SHA aneurysms were included. In the patients with acute ruptured aneurysms, non–stent-assisted procedures including simple or balloon-assisted coiling were used preferentially. Stents were applied in the cases of unstable coils mass or coils loops protruding into the parent vessel during the procedure. For unruptured aneurysms, stent-assisted coiling was typically considered first. Balloon-assisted or simple coiling was used for aneurysms with narrow necks and large dome-to-neck ratios as well as in patients who were contraindicated for antiplatelet agents. Besides, patients who presented with acute hydrocephalus all received external ventricular drainage (EVD) insertion.

All patient medical records, initial and follow-up angiograms, aneurysm characteristics, as well as aneurysm recurrence and re-treatment and EVT procedure data were reviewed retrospectively. The study was approved by the Institutional Review Board of Chang Gung Medical Foundation (IRB No: 201901382A3C502).

### EVT and follow-Up

**Figure 1:**
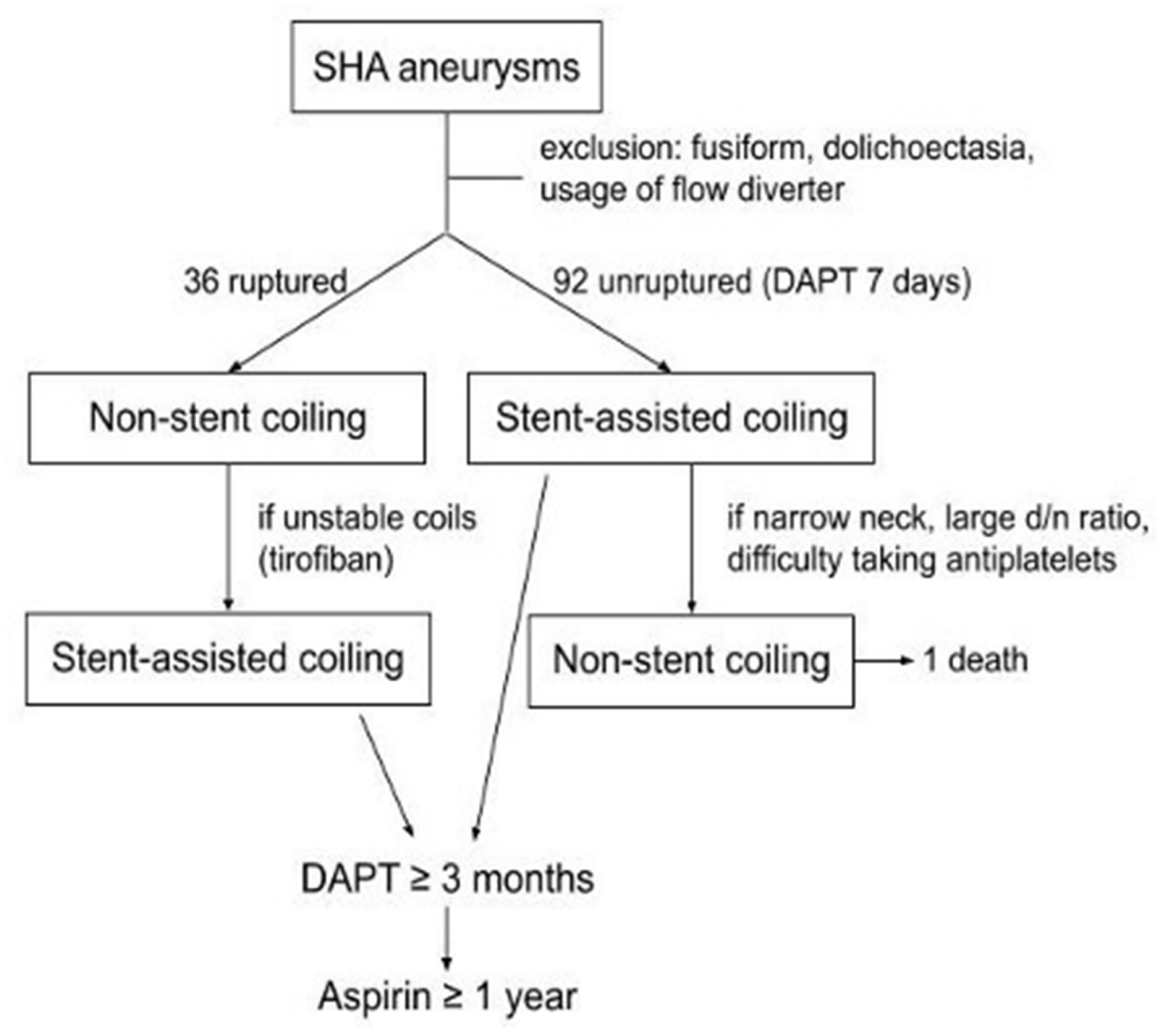
Flow of SHA aneurysm inclusion and treatment.

Embolization procedures were performed under general anesthesia by 4 experienced neurointerventionalists. All patients with unruptured aneurysm were treated preoperatively with a dual antiplatelet regimen comprising aspirin (100 mg) and clopidogrel (75 mg) daily for 7 days before the intervention. In patients with acute ruptured aneurysms, if the stent was necessary intraoperatively, tirofiban (a glycoprotein IIb/IIIa inhibitor) was immediately administered intraarterially and continuously through a slow venous drip for 24 h. In patients with deployed stents, continual dual antiplatelet therapy was advised for at least 3 months postoperatively, followed by single-agent maintenance for at least 1 year. The clinical treatment strategy is summarized in Figure 1.

**Figure 2:**
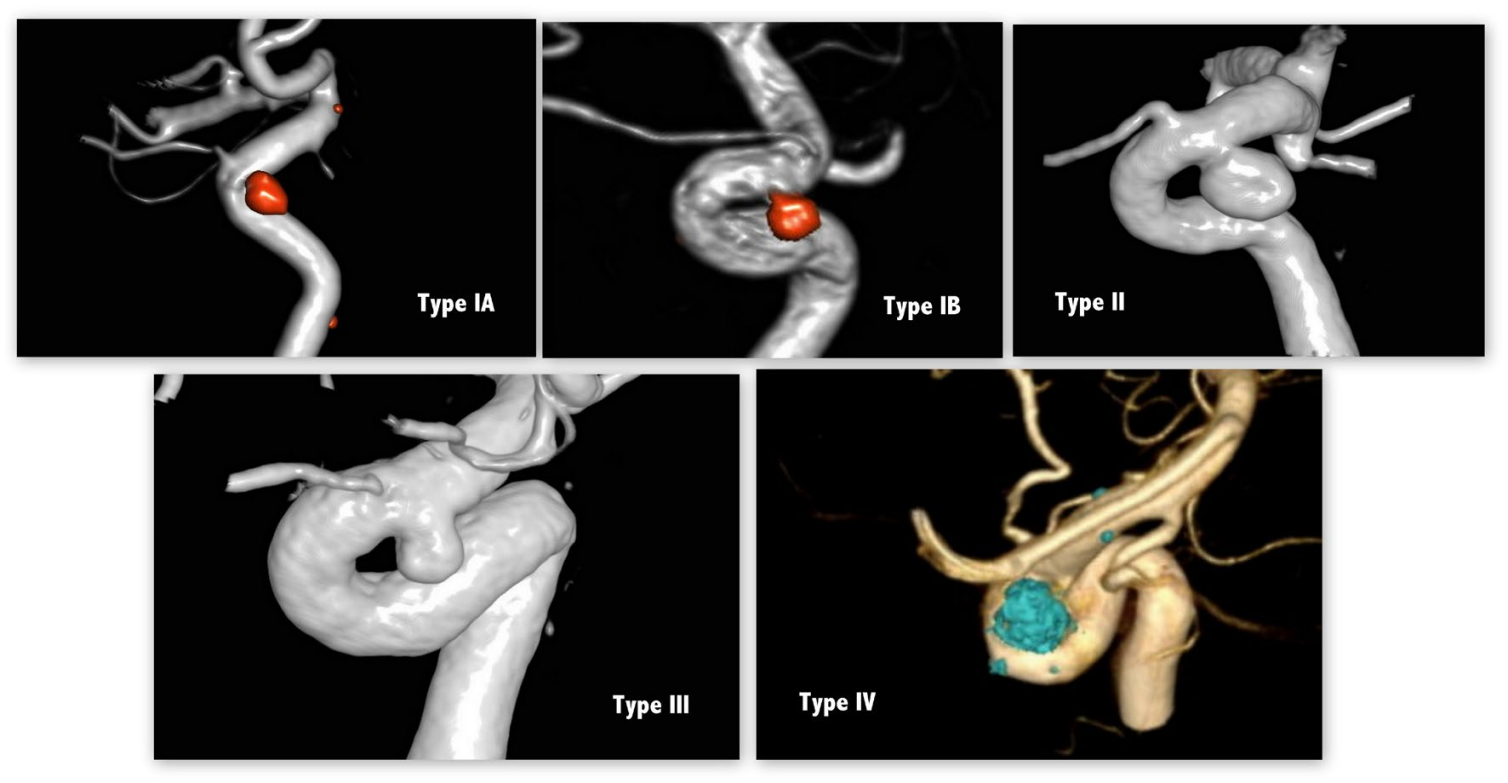
cICA tortuosity based on the angles of anterior and posterior genus.

Initial computed tomography angiography (CTA), digital subtraction angiography (DSA), and postembolization angiography data were reviewed. Cavernous ICA (cICA) was classified into 4 types according to its tortuosity—based on the angle of the anterior and posterior genus (12) —as shown in Figure 2. A type I cICA has open angles of the genus; it is subcategorized into type IA and IB when its posterior genu angle is >90° and 90°, respectively. A type II cICA is more closed than a type I cICA; in particular, the angle of a type II cICA’s anterior genu is more acute. A type III cICA exhibits posterior deflection in its posterior genu. Finally, a type IV cICA is the most tortuous with the posterior genu buckled more superiorly than the anterior genu.

Initial postembolization outcomes were classified as follows: complete occlusion (Raymond–Roy class I), when the aneurysmal sac and neck were tightly packed, and nearly complete occlusion (Raymond–Roy class II), when the sac was packed but a small neck remained.

Radiologic outcome data were obtained through conventional DSA at least 1 year after first treatment and through magnetic resonance angiography (MRA) annually in the subsequent years if the previous DSA had shown no evidence of recurrence. On the other hand, DSA were arranged when MRI had suspected recurrence. The data were grouped according to the angiographic follow-up duration (Table 1). Aneurysm recurrence was defined as regrowth after previous complete obliteration or enlargement of a previous residual neck in the subsequent image. The recurrence was considered major if it was saccular and its size theoretically permitted re-treatment with coils. Major recanalization and marked residual sac flow were the basis for repeat embolization. Stent-assisted coiling was afforded the first priority as the re-treatment modality. Clinical follow-up data were collected for all patients at least until one-and-a-half years post-embolization (except in 1 patient who died of concurrent ruptured arteriovenous malformation [AVM]), and favorable outcome was defined as modified Rankin Scale (mRS) score ≤ 2 (Table 2).

**Table 1:** Angiography follow-up for 127 EVT sessions.

	Total	1 year	2 years	3 years	>5 years
Simple coiling	7	7 (100%)	4 (57.1%)	3 (42.9%)	1 (14.3%)
Balloon-assisted coiling	48	48 (100%)	29 (60.4%)	20 (41.7%)	12 (25%)
Non-stent-assisted coiling (simple + balloon coiling)	55	55 (100%)	33 (60%)	23 (41.8%)	13 (23.6%)
Stent-assisted coiling	72	72 (100%)	36 (50%)	24 (33.3%)	10 (13.9%)
Total	127	127 (100%)	69 (54.3%)	47 (37.0%)	23 (18.1%)

Follow-up protocol was described in the Materials and Methods section.

**Table 2:** Clinical outcome of SHA aneurysms after endovascular coiling.

	Total	3 months	6 months	1 year	>1.5 year
Ruptured aneurysms	36	36 (100%)	36 (100%)	36 (100%)	36 (100%)
Favorable outcome (mRS 0–2)		28 (77.8%)	29 (80.6%)	32 (88.9%)	32 (88.9%)
Unfavorable outcome (mRS 3–5)		8 (22.2%)	7 (19.4%)	4 (11.1%)	4 (11.1%)
Mortality (mRS 6)		0	0	0	0
Unruptured aneurysms	91	91 (100%)	91 (100%)	91 (100%)	91 (100%)
Favorable outcome (mRS 0–2)		88 (96.7%)	88 (96.7%)	88 (96.7%)	88 (96.7%)
Unfavorable outcome (mRS 3–5)		3 (3.3%)	3 (3.3%)	3 (3.3%)	3 (3.3%)
Mortality (mRS 6)		0	0	0	0

mRS, modified Rankin Scale.

### Statistical analysis

All statistical analyses were performed on SPSS 25. Patient demographics, aneurysm features, and treatment characteristics were compared according to rupture by using independent *t* and chi-square tests, and the results were exhibited in Table 3. To examine whether patient characteristics and aneurysm features correlated with rupture, ruptured and unruptured SHA aneurysms were compared on the basis of patient sex, age, aneurysmal size, dome-to-neck ratio, multiplicity, and cICA tortuosity (Table 4). Treatment options and Raymond–Roy classes were added as independent variables in the recurrence and re-treatment analysis (Table 5 and Supplementary material, respectively). Variables with *p* < 0.2 in the univariate logistic regression analysis were further analyzed through multivariate logistic regression. Admittedly, multiple testing should be corrected when facing multiple comparisons problem. Nevertheless, we consider that this adjustment may not be best suited in the present study. Multiple testing will probably fix false positive problem (Type I error), but it also increases the false negative rate (Type II error). That is, many significant results may turn into insignificant, but those insignificant findings may not be statistically correct. This phenomenon is most evident when the number of comparisons is high, which was the situation in our series. Moreover, VIF (variance inflation) have been examined for all independent variables analyzed in the multiple regression analyses, and none of them were above 10. This means the multicollinearity problem did not occur in our analysis. Therefore, we believe the current statistical analyses were acceptable and reasonable for the present study. All statistical analyses were supervised by data analysts in Chang Gung Memorial Hospital Center for Big Data Analysts and Statistics. Notably, stent-assisted coiling was barely used for ruptured aneurysms because antiplatelet use is contraindicated to patients with intracranial hemorrhage; in these patients, simple coiling or balloon-assisted coiling was adopted.

**Table 3:** Patient demographics, aneurysm features and treatment characteristics.

(*N* = 127)		Ruptured (*n* = 36)	Unruptured (*n* = 92)	*P*-value
Patient factor	Female (%)	25 (69.4%)	75 (81.5%)	0.11
	Age (year)	53.17 ± 12.84	52.17 ± 11.09	0.66
Aneurysm factor	Recurrence (%)	11 (31.4%)	6 (6.5%)	<0.001
	Maximal diameter			0.37
	<3 mm (%)	4 (11.1%)	22 (23.9%)	
	3–7 mm (%)	21 (58.3%)	58 (63.0%)	
	>7 mm (%)	11 (30.6%)	12 (13.0%)	
	Dome/Neck ratio	1.71 ± 0.99	1.40 ± 0.38	0.01
	cICA type			<0.001
	Type IA (%)	24 (66.7%)	34 (37.0%)	
	Type IB (%)	11 (30.6%)	27 (29.3%)	
	Type II-IV (%)	1 (2.8%)	31 (33.7%)	
	Multiple	12 (33.3%)	29 (31.5%)	0.84
Treatment factor	Stent usage (%)	7 (19.4%)	65 (70.7%)	<0.001
	Raymond class II (%)	13 (36.1%)	23 (25.0%)	0.21

1 ruptured aneurysm was not included for recurrence analysis due to dying of concomitant ruptured AVM.

**Table 4:** Correlation factors for SHA aneurysm rupture.

				Univariate analysis		Multivariate analysis	
(*N* = 128)		Rupture (*n* = 36)	No rupture (*n* = 92)	OR (95% CI)	*P*-value	OR (95% CI)	*P*-value
Patient factor	Female (%)	25 (69.4%)	75 (81.5%)	0.52 (0.21–1.25)	0.14	0.49 (0.18–1.31)	0.15
	Age (year)	53.17 ± 12.84	52.17 ± 11.09	1.01 (0.97–1.04)	0.66		
Aneurysm factor	Maximal diameter						
	<3 mm	4 (11.1%)	22 (23.9%)	0.20 (0.05–0.76)	0.02	0.39 (0.08–2.05)	0.27
	3–7 mm	21 (58.3%)	58 (63.0%)	0.40 (0.15–1.03)	0.06	0.78 (0.24–2.52)	0.68
	>7 mm	11 (30.6%)	12 (13.0%)	1.00 (reference)		1.00 (reference)	
	Dome/Neck ratio	1.71 ± 0.99	1.40 ± 0.38	2.24 (1.10–4.57)	0.03	1.72 (0.76–3.94)	0.20
	cICA type						
	Type IA	24 (66.7%)	34 (37.0%)	21.88 (2.79–171.47)	0.003	17.37 (2.17–139.21)	0.007
	Type IB	11 (30.6%)	27 (29.3%)	12.63 (1.53–104.29)	0.02	11.60 (1.37–98.41)	0.03
	Type II-IV	1 (2.8%)	31 (33.7%)	1.00 (reference)		1.00 (reference)	
	Multiple	12 (33.3%)	29 (31.5%)	1.09 (0.48–2.47)	0.84		

**Table 5:** Risk factors for post-EVT SHA aneurysm recurrence.

				Univariate analysis		Multivariate analysis	
(*N* = 127)		Recurrence (*n* = 17)	No recurrence (*n* = 110)	OR (95% CI)	*P*-value	OR (95% CI)	*P*-value
Patient factor	Female (%)	11 (64.7%)	88 (80%)	0.46 (0.15–1.38)	0.16	1.82 (0.38–8.72)	0.45
	Age (year)	47.47 ± 12.36	53.28 ± 11.34	0.96 (0.91–1.00)	0.06	0.95 (0.89–1.01)	0.10
Aneurysm factor	Ruptured (%)	11 (64.7%)	24 (21.8%)	6.57 (2.20–19.59)	0.001	8.59 (1.36–54.22)	0.02
	Maximal diameter						
	<3 mm (%)	6 (35.3%)	20 (18.2%)	2.00 (0.44–9.13)	0.37	6.71 (0.63–71.80)	0.12
	3–7 mm (%)	8 (47.1%)	70 (63.6%)	0.76 (0.19–3.14)	0.70	0.44 (0.07–3.03)	0.41
	>7 mm (%)	3 (17.6%)	20 (18.2%)	1.00 (reference)		1.00 (reference)	
	Dome/Neck ratio	1.58 ± 0.68	1.48 ± 0.62	1.25 (0.62–2.54)	0.53		
	cICA type						
	Type IA (%)	7 (41.2%)	50 (45.5%)	2.10 (0.41–10.78)	0.37	0.50 (0.05–4.97)	0.55
	Type IB (%)	8 (47.1%)	30 (27.3%)	4.00 (0.78–20.42)	0.10	1.59 (0.20–12.46)	0.66
	Type II-IV (%)	2 (11.8%)	30 (27.3%)	1.00 (reference)		1.00 (reference)	
	Multiple	6 (35.3%)	35 (31.8%)	0.86 (0.29–2.50)	0.78		
Treatment factor	Stent usage (%)	1 (5.9%)	71 (64.5%)	0.03 (0.004–0.27)	0.001	0.03 (0.003–0.38)	0.01
	Raymond class II (%)	9 (52.9%)	26 (23.6%)	3.64 (1.27–10.38)	0.02	1.44 (0.33–6.39)	0.63

## Results

This study included 112 patients with SHA aneurysms who underwent a total of 128 EVT treatment sessions between January 2009 and December 2020; however, 1 patient who died due to concurrent ruptured AVM was not included in further analyses.

### Follow-up outcome

Table 1 presents the summary of angiographic follow-up data for all EVT sessions. In all the included patients, the follow-up duration was at least 1 year, with it being >5 years in almost one-fifth of the patients; moreover, the average follow-up length was 3.12 years. Considering the long-term follow-up duration, the current conclusions may be substantial. Table 2 compares ruptured and unruptured cohorts by clinical outcome (mRS, modified Rankin Scale) at 3 month, 6 month, 1 year and > 1.5 year. All coiled aneurysms received clinical follow-up for at least 1.5 year. At one-year postprocedure, unfavorable outcome (mRS score ≥ 3) was observed in only 4 out of 36 ruptured aneurysms. With regard to 91 unruptured aneurysms, 3 treatment sessions resulted in unfavorable outcome: one was associated with underlying parkinsonism, one resulted from intraoperative rupture followed by large infarction, and the other case came from previous MCA ruptured aneurysm.

### SHA aneurysm rupture

Table 3 compares the patient demographics, aneurysm features, and treatment characteristics between ruptured and unruptured aneurysms. Recurrence status, dome-and-neck ratio, cavernous ICA tortuosity and stent deployment rate were statistically different between the two groups (*p* < 0.05). Notably, incomplete obliteration rate (i.e., Raymond class II) did not differ between the two groups. Table 4 lists the correlation factors for SHA aneurysm rupture in our patients. In total, our patient series had 36 ruptured SHA aneurysms (28.13% [36/128]). The multivariate logistic regression analysis results showed that cICA tortuosity was associated with aneurysm rupture. The open angles of the genus (type IA and IB) were significantly correlated with aneurysm rupture: SHA aneurysms with type IA and IB cICA were 17.37 and 11.60 times more likely to rupture than were those with type II to IV cICA, respectively (*p* = 0.007 and *p* = 0.03, respectively). SHA aneurysm size was more or less correlated with aneurysm rupture risk. Large (>7-mm) and medium-sized (3-7-mm) SHA aneurysms were more likely to rupture than were small (<3-mm) SHA aneurysms (*p* = 0.27 and *p* = 0.68, respectively). However, patient age, sex, multiplicity, and dome-to-neck ratio were not significantly associated with SHA aneurysm rupture.

### SHA aneurysm recurrence

Table 5 lists the risk factors for post-EVT SHA aneurysm recurrence. Recurrence occurred for 17 SHA aneurysms (recurrence rate = 13.4% [17/127]). The recurrence rate was even lower for stent-assisted coiling (1.4% [1/72]). Notably, all recurrent aneurysms occurred within 2 years of embolization; in other words, even if residual neck presented, the sacs did not enlarge after 2 years after embolization.

Moreover, aneurysm rupture and non–stent-assisted coiling were found to be independent risk factors for aneurysm recurrence. Aneurysm recurrence was 8.59 times more likely to occur for ruptured aneurysms than for unruptured aneurysms (*p* = 0.02). Furthermore, stent-assisted coiling was a negative contributor for post-EVT recurrence. Stent-assisted coiling led to 3% recurrence rate that of non–stent-assisted coiling (*p* = 0.01). Patient age, sex, dome-to-neck ratio, cICA tortuosity, multiplicity, and Raymond–Roy class did not affect recurrence significantly.

### SHA aneurysm re-treatment

Of the 17 recurrent aneurysms, 9 were re-treated (re-treatment rate = 7.1% [9/127]; Supplementary material). In patients who received stent-assisted coiling, the re-treatment rate was notably lower (1.4% [1/72]). In the current study, rupture occurrence was the only risk factor that correlated with SHA aneurysm re-treatment. Ruptured aneurysms were 13.66 times more likely to receive re-treatment than were unruptured aneurysms (*p* = 0.04). However, patient sex, age, aneurysm size, dome-to-neck ratio, cICA tortuosity, multiplicity, stent usage, and Raymond–Roy class did not affect the odds of re-treatment significantly. No more recurrence occurred after retreatment with stent-assisted coiling.

Regarding EVT-related complications in patients with SHA aneurysms, two instances of intraoperative complications (1 bleeding and 1 thrombus formation) occurred in the 128 embolization sessions. In the case with acute thrombosis, immediate thrombectomy was performed, and no postoperative sequela was noted. Postembolization stroke occurred in 5 cases (3 transient ischemic attacks and 2 infarctions). Moreover, intracerebral hemorrhage (ICH) occurred in 1 patient, but it was not due to re-bleeding but suspectedly due to spontaneous hemorrhage caused by coagulopathy resulting from liver cirrhosis.

## Discussion

Previous studies have categorized SHA aneurysms as paraclinoid aneurysms (1, 3, 4), which are considered to have a low rupture rate (5, 6). However, consistent with the results of Chalouhi, Tjoumakaris (7), rupture occurred in a large proportion (28.1%) of our SHA aneurysm cases. Furthermore, subarachnoid hemorrhage caused by aneurysm rupture can not only lead to high mortality rate but result in high degree of disability (8). Considering their tendency to rupture and result in SAH, SHA aneurysms should be treated actively, as early as possible so as to prevent the detrimental clinical outcomes and its associated recurrence and re-treatment.

In the present study, all patients had been angiographically followed up for at least 1 years, with the average follow-up duration being 3.12 years. Therefore, the results of the current EVT series for SHA aneurysms are highly relevant and valid. Furthermore, because the main focus of this study was saccular SHA aneurysms with EVT, we excluded fusiform aneurysms and aneurysms with dolichoectasia; these variations may represent another disease entity, which may require different treatment strategy, such as the use of flow-diverter. In addition, the use of flow-diverter was excluded in the current study because it accounted for a limited proportion of our treatment sessions because of our national insurance coverage’s limitation and a high number of ruptured cases in our series.

In general, cICAs can be divided into 4 types based on their tortuosity (12). In our study, SHA aneurysms with less tortuous cICA (i.e., type IA and IB cICA) tended to rupture. This result, which has not been reported thus far, may occur due to a direct bloodstream flowing into the aneurysm sac. Computational fluid dynamics, which can simulate dynamic blood flow of intracranial aneurysms, has been widely adopted to investigate the contributing factors for aneurysm rupture (13). Aneurysmal hemodynamics influence the rupture risk of paraclinoid aneurysms (14). Moreover, inflow angle—the angle between the aneurysm dome axis and the parent vessel—has been found to affect aneurysmal hemodynamics. An increased inflow angle increases peak velocity and energy transmission to the dome, followed by the achievement of a discriminate rupture status (15). Although the association between cICA tortuosity and fluid dynamics remains unknown, we hypothesized that the tortuosity of cICA is associated with the peak velocity and energy transmission of blood flow to the aneurysm sac based on the above findings. cICAs with an obtuse angle (i.e., type IA and IB cICAs) ease the blood flow into the aneurysm sac, whereas more tortuous cICAs (i.e., type II-IV cICAs) prevent this blood flow. Therefore, treating SHA aneurysms in less-curved cICAs require procedures such as stent-assisted coiling because they afford a relatively low rupture risk (16). Admittedly, additional flow dynamics studies to explore the association between vessel tortuosity and fluid mechanics are warranted.

In this study, large (>7-mm) aneurysms were to some extent associated with rupture status; this result corroborates that of previous studies (17–19): The International Study of Unruptured Intracranial Aneurysms (ISUIA) trial reported that the 5-year cumulative rupture rate was considerably lower in small (<7-mm) unruptured aneurysms, regardless of anterior or posterior circulation (19). A 2009 review also identified a size of >7-mm as independent risk factor for aneurysm rupture (18). Finally, in their long-term follow-up study with an average of 18.5-year follow-up, Korja, Lehto (17) confirmed that large (>7-mm) size is a risk factor for aneurysm rupture. As a result, large (>7-mm) aneurysms warrant timely treatment of stent-assisted coiling to prevent rupture and recurrence (16).

Large aneurysm size, aneurysm rupture, incomplete occlusion, and non-stent use have been reported to be associated with aneurysm recurrence after EVT (20–22). In the current study, aneurysm rupture and non–stent-assisted coiling, but not large size or incomplete occlusion, demonstrated similar effects on SHA aneurysms. This may be because large aneurysms accounted for only a small proportion (18.1%) of our data, and few (27.6%) of our patients demonstrated incomplete occlusion (Raymond–Roy class II or III). In contrast to angiography results reported elsewhere (21), most (72.4%) aneurysms in the current study achieved complete occlusion, possibly because of technological advancement and technical improvements since the aforementioned study was reported. In addition, compared with unruptured aneurysms (25% [23/92]; Table 2), incomplete occlusion rate was comparable in ruptured aneurysms (36.1% [13/35]; *p* = 0.21). Therefore, the claim that higher recurrence rate in ruptured aneurysms results from loose coils compaction in the fear of thromboembolism when antiplatelet therapy is contraindicated is unsubstantiated.

Consistent with previous findings (7), the post-EVT SHA aneurysm recurrence rate (13.4% in our series) was equal to or lower than that in other locations, reported elsewhere: the recurrence rates for aneurysms of the A-com artery (23), MCA (24), P-com artery (21), and posterior circulation (25) have been reported to be 14.6, 20, 37, and 24.5%, respectively. The relative lower recurrence rate highlights the satisfactory outcome of coils embolization and the importance of initial treatment for SHA aneurysms. Moreover, when recurrent SHA aneurysms were re-treated with stent-assisted coiling, no further recurrence or rebleeding occurred until the last follow-up. Therefore, SHA aneurysms can be reliably secured with stent use. Nonetheless, these findings require confirmation by studies with a longer follow-up duration and larger sample size.

Flow diverter is now considered an effective treatment tool for the management of complicated intracranial aneurysms that are difficult to treat with coiling and microsurgery (26, 27). Compared with coiling technique, it provides a higher complete occlusion rate and lower recurrence rate (28) because it can secure aneurysm sac more firmly. Although it seems an appropriate treatment modality for SHA aneurysms, flow-diverter treatment was excluded in the study. As a result, direct comparison of treatment outcomes between coiling and flow-diverter could not be achieved in the present study. However, when flow diverters are not amenable or suitable, stents can still offer satisfactory results in the SHA aneurysm treatment provided the results of the current study. Therefore, stent-assisted coiling is an reasonable treatment option for SHA aneurysms, in particular when flow diverters are not accessible and available.

Stent-assisted coiling achieves a lower recurrence rate than simple coiling or balloon-assisted coiling does (16). However, according to the 2012 *Guidelines for the Management of Aneurysmal Subarachnoid Hemorrhage*, stenting of a ruptured aneurysm is associated with increased morbidity and mortality (29); this is because antiplatelet therapy is generally contraindicated in the acute settings (16). Therefore, ruptured SHA aneurysms were typically treated with non–stent-assisted coiling: in the current study, 29 (80.56% [29/36]) ruptured aneurysms were treated with non–stent-assisted coiling. Although the clinical outcome was favorable (mRS score ≤ 2) in 28 (96.55%) of the cases, their recurrence rate was relatively high (39.29% [11/28]). However, if the recurrence was detected early and treated with stent-assisted coiling, no re-recurrence was noted. Moreover, no recurring hemorrhages occurred during the long-term follow-up. This low recurrence rate after stent use is in line with that reported previously (7). The results reflect that stent-assisted coiling is effective in the recurring SHA aneurysms. Combined with the low complication rate for coils embolization in the present study, we therefore suggest early stent assistance in the treatment of ruptured SHA aneurysms.

## Limitations

This study has several limitations. First, its retrospective nature may have contributed to selection bias. Second, operator preference and patient condition may have affected the applied treatment strategy, thus altering our findings. To mitigate subjective decision bias, we based our clinical decisions on the consensus of a multidisciplinary team. Third, comprehensive risk factors, such as hypertension, diabetes, and family history, were not considered in this analysis because a considerable amount of patient data was missing. Fourth, although all patients were angiographically followed up at 1 year post-coiling, only 23 EVT treatment sessions (18.1% [23/127]) had received image follow-up after 5 years; extended follow-up period necessitates as late recanalization may still occur even after stent-assisted coiling.

Despite these limitations, our study sheds light on the characteristics of saccular SHA aneurysms and the applicable treatment strategies, especially in areas where flow diverter was not amenable. Although this study has the longest follow-up period reported to date, a relevant study with a longer follow-up duration is warranted to investigate the long-term treatment outcomes further.

## Conclusion

In this study, although flow diversion is another well-suited treatment modality for SHA aneurysms that was not compared with, EVT with stent-assisted coiling was an effective treatment modality for SHA aneurysms, with low recurrence and complication rates. Type I cICA was common factor for aneurysm rupture. In most cases, recurrence occurred within 2 years of EVT and was significantly associated with prior aneurysm rupture and non–stent-assisted coiling. Finally, further stent-assisted coiling could prevent re-recurrence.

## Data availability statement

The original contributions presented in the study are included in the article/Supplementary material, further inquiries can be directed to the corresponding author.

## Ethics statement

The studies involving human participants were reviewed and approved by the Chang Gung Medical Foundation Institutional Review Board. The patients/participants provided their written informed consent to participate in this study. Written informed consent was obtained from the individual(s) for the publication of any potentially identifiable images or data included in this article.

## Author contributions

Y-PK contributed to conception and design of the work and drafted the manuscript. C-YL performed the statistical analysis. C-TC, M-CY, P-CH, Z-HL, and C-CChu carried out techniques review and conduct. H-FW and Y-MW conducted data review and received techniques consultation. C-CChe undertook final review and interpretation. All authors contributed to manuscript revision, read, and approved the submitted version.

## Conflict of interest

The authors declare that the research was conducted in the absence of any commercial or financial relationships that could be construed as a potential conflict of interest.

## Publisher’s note

All claims expressed in this article are solely those of the authors and do not necessarily represent those of their affiliated organizations, or those of the publisher, the editors and the reviewers. Any product that may be evaluated in this article, or claim that may be made by its manufacturer, is not guaranteed or endorsed by the publisher.
