# Supplementary material for: Long-term outcomes of coils embolization for superior hypophyseal artery aneurysms
Source: Front Neurol. 2023 Jun 29;14:1096970. doi: 10.3389/fneur.2023.1096970 (PMC10338873; doi:10.3389/fneur.2023.1096970)
Supplement: Supplementary file 1 [file Data_Sheet_1.docx]

**Supplementary Material. Risk factors for post-EVT SHA aneurysm re-treatment**

|  |  |  |  | Univariate analysis | | Multivariate analysis | |
| --- | --- | --- | --- | --- | --- | --- | --- |
| (*N*=127) |  | Retreatment (*n*=9) | No retreatment (*n*=118) | OR (95% CI) | *P*-value | OR (95% CI) | *P*-value |
| Patient factor | Female (%) | 4 (44.4%) | 95 (80.5%) | 0.19 (0.05-0.78) | 0.02* | 0.39 (0.06-2.34) | 0.30 |
|  | Age (year) | 43.44 ± 10.29 | 53.19 ± 11.44 | 0.92 (0.86-0.99) | 0.02* | 0.94 (0.87-1.02) | 0.12 |
| Aneurysm factor | Ruptured (%) | 8 (88.8%) | 27 (22.9%) | 26.96 (3.23-225.25) | 0.002* | 13.66 (1.20-155.98) | 0.04* |
|  | Maximal diameter |  |  |  |  |  |  |
|  | <3mm (%) | 1 (11.1%) | 25 (21.2%) | 1.00 (reference) |  |  |  |
|  | 3-7mm (%) | 6 (66.6%) | 72 (61.0%) | 2.08 (0.24-18.16) | 0.51 |  |  |
|  | >7mm (%) | 2 (22.2%) | 21 (17.8%) | 2.38 (0.20-28.14) | 0.49 |  |  |
|  | Dome/Neck ratio | 1.75 ± 0.88 | 1.47 ± 0.61 | 1.60 (0.74-3.44) | 0.23 |  |  |
|  | cICA type |  |  |  |  |  |  |
|  | Type IA (%) | 6 (66.6%) | 51 (43.2%) | N/A | N/A |  |  |
|  | Type IB (%) | 3 (33.3%) | 35 (29.7%) | N/A | N/A |  |  |
|  | Type II-IV (%) | 0 | 32 (27.1%) | 1.00 (reference) |  |  |  |
|  | Multiple (%) | 1 (11.1%) | 40 (33.9%) | 0.24 (0.03-2.02) | 0.19 | 0.21 (0.02-2.42) | 0.21 |
| Treatment factor | Stent usage (%) | 1 (11.1%) | 71 (60.2%) | 12.09 (1.46-99.81) | 0.02* | 0.33 (0.03-4.28) | 0.39 |
|  | Raymond class II (%) | 5 (55.5%) | 30 (25.4%) | 3.67 (0.92-14.55) | 0.07* | 2.13 (0.34-13.51) | 0.42 |
